# Risk Assessment Instruments for Intimate Partner Femicide: A Systematic Review

**DOI:** 10.3389/fpsyg.2022.896901

**Published:** 2022-05-31

**Authors:** Esperanza Garcia-Vergara, Nerea Almeda, Francisco Fernández-Navarro, David Becerra-Alonso

**Affiliations:** ^1^Departament of Quantitative Methods, Universidad Loyola Andalucia, Seville, Spain; ^2^Departament of Psychology, Universidad Loyola Andalucia, Seville, Spain

**Keywords:** intimate partner violence, homicide, prediction, risk assessment, systematic review, women

## Abstract

Intimate partner violence is a severe problem that has taken the lives of thousands of women worldwide, and it is bound to continue in the future. Numerous risk assessment instruments have been developed to identify and intervene in high-risk cases. However, a synthesis of specific instruments for severe violence against women by male partners has not been identified. This type of violence has specific characteristics compared to other forms of intimate partner violence, requiring individualized attention. A systematic review of the literature has been conducted to summarize the intimate partner homicide risk assessment instruments applied to this population. It has been carried out with the Preferred Reporting Items for Systematic Reviews and Meta-Analyses statement guidelines. The search strategy yielded a total of 1,156 studies, and only 33 studies met eligibility criteria and were included in the review. The data of these studies were extracted, analyzed, and presented on study characteristics (country and year, sample, data sources, purpose of the studies) and main findings (a brief description of the instruments, risk factor items, psychometric properties). The results indicate that the Danger Assessment, the Danger Assessment for Immigrants, the Danger Assessment for Law Enforcement, the Danger Assessment-5, the Taiwan Intimate Partner Violence Danger Assessment, the Severe Intimate Partner Risk Prediction Scale, The Lethality Screen, and the H-Scale are specific risk assessment instruments for predicting homicide and attempted homicide. There are differences in the number and content of risk assessment items, but most of them include the evidence's critical factors associated with homicide. Validity and reliability scores of these instruments vary, being consistency and accuracy medium-high for estimating homicide. Finally, implications for prediction and prevention are noted, and future research directions are discussed.

## 1. Introduction

Violence against women is a global health problem of epidemic proportions (World Health Organization, 2013, 2021). It is estimated that around 35% of women suffer violence in their lifetime, being the most common violence type perpetrated by intimate partners, which affects approximately 30% of females worldwide (Devries et al., 2013). Thus, a woman is more likely to be injured, raped, or killed by her intimate partner than other people (World Health Organization, 2005; United Nations, 2006). There are different terms to refer to this violence, such as domestic violence, violence against women, intimate partner violence, marital violence, or wife assault. The denomination “partner violence against women” (PVW) is used in the current study. It refers to a physical, sexual, and psychological assault against women from their current or former men partners (Cunha and Goalves, 2016; Spencer and Stith, 2020).

Victimization has negative consequences for women's health, causing injuries and anxiety-stress responses that affect the gastrointestinal, cardiac, reproductive, and neurological systems (Wisner et al., 1999; Ruiz-Pérez et al., 2007; Ellsberg et al., 2008). Irreversible and permanent cessation of vital functions of the organism causing death is the most severe outcome of PVW (Snider et al., 2009). It affects 30 thousand women's lives annually, constituting approximately 40% of all homicides (Stöckl et al., 2013; United Nations Office on Drugs and Crime, 2018). The term “intimate partner femicide” (IPF) is used in this study when referring to severe violence resulting in women's deaths that are committed by their present or former intimate partners in heterosexual relationships (Campbell et al., 2009; Storey and Hart, 2014; Messing et al., 2015a; Messing and Campbell, 2016).

The high rates and severity of PVW highlight the importance of predicting future violence, based on scientific evidence, to manage this risk (Messing et al., 2020b). This prediction is possible through the development of risk assessment instruments that evaluate the danger in the violent relationship and take the information for aggressors' intervention and victim's safety planning (Campbell et al., 2009; Echeburúa et al., 2009). Numerous risk assessment instruments exist to assess different outcomes related to intimate partner violence as physical and sexual violence, recidivism or reassault, and homicide (Dutton and Kropp, 2000; Hanson et al., 2007; Kropp, 2008; Nicholls et al., 2013; Graham et al., 2019). Tools with the power to predict homicide are required to discern between potential lethal and non-lethal violence cases. Scientific evidence reveals that there are notable differences in victim, offender and situational characteristics that contribute to the probability of PPW escalating to IPF (Jung and Stewart, 2019; Overstreet et al., 2021). The increase in frequency and severity of violence, separation/divorce and kill threats are some of the main factors on which research in the field agree (Nicolaidis et al., 2003; Belfrage and Rying, 2004; Campbell et al., 2009; Dobash and Dobash, 2011; Kivivuori and Lehti, 2012; Vatnar and Bjørkly, 2013; Cunha and Goncalves, 2016; Johnson et al., 2020; Monckton Smith, 2020; Abrunhosa et al., 2021). It is the occurrence of certain elements that lead to IPF, not a simple progression of violence (Dobash et al., 2007). This information assists professionals to effectively manage the limited resources available by focusing their efforts on quickly, comprehensively, and effectively protecting those victims who are at high risk of being killed by their intimate partners (Storey and Hart, 2014).

Risk assessment instruments refer to tools that assist the “decision-making process through which we have to determine the best course of action by estimating, identifying, qualifying, or quantifying risk” (Nicholls, 2006). In this context, the risk is understood as the probability that an individual will engage in a certain kind of behavior in the future (Otto and Douglas, 2011; Fedock and Covington, 2019). Hence, specialized risk assessment tools designed for intimate partner homicide assess the risk of a lethal assault perpetrated by one partner against the other.

Most of the intimate partner homicide risk assessment instruments are applied regardless of the sex of both victim and aggressor (men to women, women to men, men to men, and women to women relationships) (Nicholls et al., 2013; Graham et al., 2019). PVW is an entirely different category of violence from other forms of intimate partner violence because it is the manifestation of a historical gender asymmetry and unequal power in relationships between men and women that led to domination and subordination (Russell and Harmes, 2001; De Jesus and da Silva, 2018). Men use violence as a demonstration to women that they have the authority in the relationship, having women controlled and subjected to their criteria (Anderson, 2005; González and Rodríguez-Planas, 2020). As violence, in this case, is different from those happening in other types of relationships, this phenomenon needs to be considered when using sensitive risk assessment instruments.

The inexistence of a gold standard in homicide risk assessment instruments for the diverse groups of intimate partner relationships could lead to errors in predictions (Nicholls et al., 2013). Deaths are predictable and preventable if adequate tools are used to target the population-based on factors known associated with it in each case (Johnson et al., 2019). Thus, for the prediction of IPF, it is essential to use tools that include risk factors items specific to female victimization by male aggressors in relationships. These might not coincide with the most convenient tools for intimate partner homicide in general. Summarizing sensible risk assessment instruments for IPF is a priority because of its high prevalence compared with homicide in other intimate partner relationships groups (Garcia et al., 2007; Messing et al., 2013). However, no studies focusing on this aspect have been identified to date. Graham et al. (2019) identified the need for future research to assess the reliability, validity, and feasibility of intimate partner violence and homicide risk assessment instruments in the diverse intimate relationship population.

Reliability and validity are common psychometric properties used to evaluate intimate partner homicide and reassault risk assessment instruments (Graham et al., 2019). Reliability refers to the reproducibility or consistency of measurement tools in obtaining the same results on repeated application to a person or group under similar circumstances (Cook and Beckman, 2006). The procedures most used to determine reliability are internal consistency reliability and interrater reliability (Heale and Twycross, 2015; Graham et al., 2019). The first refers to the degree to which the different items of an instrument perform together to measure a construct consistently, using the Cronbach's α commonly. In the area of violence risk assessment, Semahegn et al. (2019) indicated that the minimum acceptable value of the Cronbach's α is 0.7 (Nunnally, 1994; DeVellis, 2003; Kimberlin and Winterstein, 2008; Taber, 2018). Interrater reliability analyses consistency under agreement responses among multiple raters on different items of an instrument, using standard statistics of percentage agreement, interclass and intraclass correlation, Pearson's *r*, Spearman's *p*, and Cohen's *k* (Nunnally, 1994; DeVellis, 2003). Intraclass correlation coefficient is widely used and, in the field of violence risk assessment, Telles et al. (2009) mentioned that values from 0.4 to 0.6 are acceptable.

Validity refers to the degree of accuracy of an instrument in measuring the theoretical construct that it is intended to measure, revealing whether it can be used for its intended purpose (Kimberlin and Winterstein, 2008; Sampieri, 2018). There are different types of validity, including content, construct, and criterion validity. The content validity is concerned with the extent to which the substance of the instrument's elements is adequate to assess the specific domains that encompass the construct measured (Carmines and Zeller, 1979). The construct validity refers to how well an instrument reflects and measures a theoretical concept by determining the strength of correlation between the components of the instrument to know they are parts of the exact theoretical concept measurement and differ from other measures (Cronbach and Meehl, 1955). The criterion validity is based on a comparison of the instrument with another external criterion that measures the same construct through correlations' analysis of the results obtained in them (DeVellis, 2003). If all criteria types apply at the same time, the validity is said to be concurrent (Kaplan and Saccuzzo, 2013).

Predictive validity is a specific form of criterion validity, and it refers to the accuracy of an instrument for predicting a future criterion measure such as homicide (Messing et al., 2013, 2015a). This point is typically assessed in terms of sensitivity, specificity, positive predictive value (PPV), negative predictive value (NPV), Receiver Operating Characteristic (ROC), and Area under the Curve (AUC). Sensitivity refers to the correct identification of cases expected to meet the predictive criterion, whereas specificity targets cases that are not expected to meet it. For instance, the sensitivity in risk assessment instruments for intimate partner homicide refers to the correct classification of lethal cases and the specificity to the correct classification of non-lethal cases (Parikh et al., 2008; Loinaz, 2017; Graham et al., 2019). PPV is the probability of cases that are expected to meet the criterion, and it occurs. NPV is the probability of cases not expected to meet the criterion, and it does not occur. Following the previous example, PPV corresponds to the probability of victims that are expected to be killed and indeed die, and NPV to the probability of victims that are not expected to be killed and do not die (Faller, 2005; Akobeng, 2007). The ROC is a graph that plots sensitivity as a function of 1-specificity obtaining the AUC. These provide information of predictive accuracy on a scale of 0 to 1. An AUC of 0.50 indicates an inability to predict. The closer to 1.0, the better the prediction accuracy (Messing and Thaller, 2013; Messing et al., 2013; Loinaz, 2017). For the field of violence risk assessment, Rice and Harris (2005) indicate that values between 0.6 and 0.7 are considered acceptable.

For the mentioned, the purpose of the current systematic review is to synthesize the scientific knowledge of risk assessment instruments used specifically for IPF, which aid in predicting cases in danger and, subsequently, the prevention of lethal results. Hence, the research questions are the following:

1. What are the specific risk assessment instruments for IPF?
2. What are the risk factors for IPF included in the instruments?
3. What are the reliability and validity of the instruments?

## 2. Method

The reporting of the current systematic review was guided by the standards of the Preferred Reporting Items for Systematic Review and Meta-Analysis (PRISMA) Statement (Page et al., 2021). It details a process of identification, selection, appraisal, and synthetization of the studies to ensure a quality scientific review. Even though this guide was primarily used in the health framework, it has been applied to other areas of research related to intimate partner violence (Gerino et al., 2018; Velotti et al., 2018). A meta-analysis has not been developed because of the heterogeneity of statistical information available on the publications included in the current study, as the findings were not comparable.

### 2.1. Search Strategy

The search strategy was conducted on November 13, 2021, in the following databases: Web of Science (WOS), SCOPUS, PROQUEST, APA PsycInfo, APA PsycArticles, and CINAHL COMPLETE. The search terms included on them were composed of three sets of keywords combined with different Boolean operators, that is (“domestic violence” OR “intimate partner violence” OR “violence against women” OR “gender-based violence” OR “spous* abuse” OR “spous* violence”) AND (“danger assessment” OR “risk tool” OR “risk assessment” OR “lethality assessment” OR “instrument” OR “evaluation” OR “appraisal”) AND (“homicide” OR “murder” OR “mortality” OR “kill” OR “lethal*” OR “severe violence” OR “femicide”). The search was limited by the mentioned terms in the title and abstract, both separately and together.

### 2.2. Eligibility Criteria

Studies were included in the systematic review if they (1) examine the available risk assessment instruments for IPF developed and tested with women victims and male offenders' samples, (2) apply these instruments in the IPF field, (3) are empirical articles, (4) are in English and (5) are accessible in full text.

Studies were excluded if they (1) explore risk assessment instruments for intimate partner homicide for mixed and same-sex partnerships, (2) analyze risk appraisal tools that predict intimate partner violence in general not including homicide, (3) examine risk instruments that evaluate interpersonal violence in unspecifying populations, (4) appraise medical assessment tools used with victims and aggressors, (5) evaluate programs, guides and protocols aim at prevention of intimate partner homicide, (6) incorporate case studies, (7) constitute a systematic review and meta-analysis, (8) are books, book chapters, and theses, (9) are not empirical studies, (10) are not English and (11) are not accessible in full text.

### 2.3. Study Selection, Data Collection, and Summary Measures

After piloting the search strategy, duplicate studies were immediately removed. Subsequently, a study selection process of the remaining ones was carried out by peers in two phases. First, researchers screened the studies by titles and abstracts following the inclusion and exclusion criteria. Second, those that passed the screening were checking a full-text read using the same criteria for eligibility. The studies that met the inclusion criteria were included, and their quality was assessed.

Data extracted were organized into five sections. First, the study selection section describes the identification, screening, eligibility, and inclusion of the studies. Second, the study characteristics section includes the country and year, sample, data sources, and purpose of the studies included in the systematic review. Third, the main findings section provides knowledge about the risk assessment instruments for IPF, including a description of each detected and their number and content of the risk factor items, reliability, and validity. Fourth, the quality of included studies section includes the quality assessment results.

### 2.4. Quality Assessment

#### 2.4.1. Quality Assessment of the Search Strategy

The design and execution of the search strategy in scientific databases is a relevant element in elaborating systematic reviews since it provides the studies that will be part of it. A quality assessment of the search strategy is essential to identify its adequacy in obtaining studies on a determined theme and, therefore, to support final results that respond to the research objectives. Hence, one of the quality indicators analyzed is the content of the studies included in the systematic review. It is assumed that if these studies' keywords and research topics respond to the research questions, the search strategy employed has been adequate.

A Latent Semantic Analysis (LSA) was also implemented in this section. LSA is a text mining methodology for extracting and deciphering key latent factors existing in initially unclustered texts (Landauer et al., 1998). LSA uses information extraction and natural language processing techniques and applies them with algorithms and methods from data mining, machine learning, and statistics (Evangelopoulos et al., 2012). The keywords of the studies included in the systematic review are extracted and transformed into a frequency matrix with the term frequency-inverse document frequency (TF-IDF) weighting method (Havrlant and Kreinovich, 2017). This weighting schema increases the relevance of uncommon keywords and reduces the usual ones by emphasizing uniqueness. After that, the singular value decomposition (SVD) technique was applied to the weighted matrix to decompose it into three matrices: (1) the term-by-factor matrix showing the loadings of keywords on a particular latent factor, (2) the singular value matrix representing the importance of certain factors, and (3) the document-by-factor matrix presenting the loadings of texts on a particular latent factor. Each latent factor is linked to specific high-loading keywords and to the text of the studies representing the same underlying research theme that makes this association possible by variance explained.

#### 2.4.2. Quality Assessment of the Studies

Examining the rigorousness of the included studies is an essential part of the review process since the evidence reported in them impacts the findings of the current study. No specific instruments have been identified to evaluate the quality of studies included in reviews related to intimate partner violence/homicide risk assessment instruments. Several systematic reviews and meta-analyses on intimate partner violence and violence risk assessment tools use criteria adapted from the Critical Appraisal Skills Programme (2020) (CASP) to assess the risk of bias within studies, and some of them combine it with another guidance from the Effective Public Practice Project (2008) (EPHPP) and Centre for Review and Disseminations (2009) (CRD) (Lagdon et al., 2014; Geraghty and Woodhams, 2015; Rossdale et al., 2020). In the current study, both themes are analyzed, which is why the mentioned checklists are appropriate to use. Specifically, the tool created by Geraghty and Woodhams (2015) which combines these three checklists is used. It includes 16 items categorized into four sections: selection bias, measurement bias, attrition bias, and reporting bias. The scoring system consists on assigning a score of 0 to each item if the conditions are not met, 1 if they are partially met, and 2 if they are entirely met (Geraghty and Woodhams, 2015). There is no cut-off score indicating high or low quality, being designated by the experts' criteria. The quality of each study was assessed by two researchers.

## 3. Results

### 3.1. Study Selection

**Figure 1:**
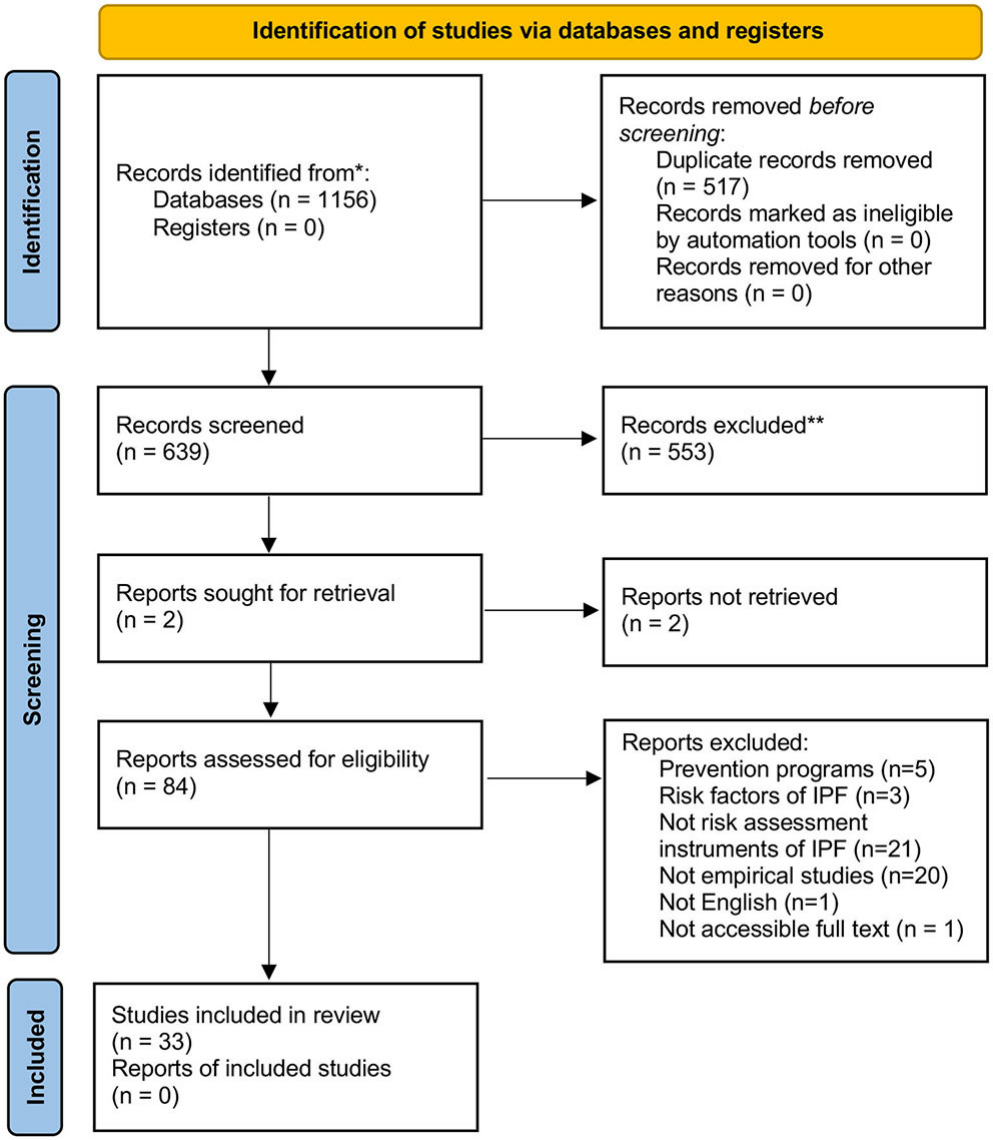
PRISMA flow diagram of the study selection process (Page et al., 2021).

The search strategy yielded a total of 1,156 publications across all databases. 517 was removed as duplicates, and 639 publications remained for screening. Of those, 84 met the inclusion criteria under the title and abstract read, and 555 of them were removed from the exclusion criteria. After a full-text reading, 51 publications were removed, and 33 were included in the systematic review according to the eligibility criteria (see Figure 1).

### 3.2. Study Characteristics

The characteristics of the included studies are summarized in the Table 1. The relevant findings are presented in the following paragraphs.

**Table 1:** Characteristics of the included studies in the systematic review.

**Studies**	**Study location**	**Sample of the study**	**Data source of the study**	**Study purpose related with the tools**
Messing et al. (2015a)	USA	254 victims of PVW	Structured interviews, the Lethality Screen (Messing et al., 2015a), the Danger Assessment (DA) (Campbell et al., 2003, 2009) and the revised Conflict Tactics Scale (CTS-2) (Straus et al., 1996)	To examine the predictive validity of the Lethality Screen
Messing et al. (2020b)	USA	959 victims of PVW and attempted IPF	Structured interviews and the DA (Campbell et al., 2003, 2009)	To develop and testing the Danger Assessment for Law Enforcement (DA-LE)
Richards et al. (2019)	USA	141 victims of PVW seeking legal aid service	The Lethality Assessment Program (LAP), the CTS2 (Straus et al., 1996), the Safety Promoting Behavior Checklist (McFarlane et al., 2004) and the Personal Progress Scale-Revised (PPS-R) (Johnson et al., 2005)	To assess whether receiving the LAP -including the Lethality screen and the Lethality Assessment Protocol- impact on women's awareness risk for severe violence or homicide and empowering to self-protective measures and seek professional services
Sabri et al. (2019)	USA	1250 immigrant, refugee, and indigenous victims of PVW	The CTS-2 (Straus et al., 1996), the Patient Health Questionnaire (PHQ-9) (Kroenke et al., 2001), the Harvard Trauma Questionnaire (Mollica et al., 1992), the PPS-R (Johnson et al., 2005), and the Measure of Victim Empowerment Related to safety (MOVERS) scale (Goodman et al., 2015)	To test the effectiveness of two cultural versions of the Safety Planning Interventions (“myPlan”) to immigrant, refugee and indigenous populations. Them are entitled “weWomen” for immigrant and refugee populations, and “OurCircle” for indigenous populations. These versions are integrated by an adapted version of DA to these populations, developing safety interventions to women victims based on the PVW and IPF risk
Glass et al. (2009)	USA	209 victims of PVW	The DA (Campbell et al., 2003)	To determine typologies of PVW/IPF survivors based on known risk factors of the phenomenon
Glass et al. (2008)	USA	53 victims of PVW and 23 victims of IPF	The DA (Campbell, 1986)	To identify risk factors of IPF in young adult population
McFarlane et al. (1998)	USA	199 pregnant victims of PVW	The Index of Spouse Abuse (ISA) (Hudson and McIntosh, 1981), the DA (Campbell, 1986), and the Severity of Violence Against Women Scale (SVAWS) (Marshall, 1992)	To examine the severity of PVW in pregnant women victims and its association with gun access by the aggressor
Dutton et al. (2018)	USA	16 professionals of social service agencies who administer LAP in PVW cases	Semi-structured interviews	To assess the experiences and perceptions of domestic violence agency professionals with the LAP
Grant and Cross-Denny (2017)	USA	22 police officers that administer LP in their departments	Focus groups	To explore the attitudes and barriers of police officers to a successful implementation of LAP
Messing et al. (2016)	USA	648 victims of PVW	Semi-structured interviews	To analyze the applications of the LAP to women victims of PVW/IPF
Johnson et al. (2020)	USA	213 women victims of PVW and attempted IPF	The DA (Campbell et al., 2009), the Abusive Behavior Inventory-Revised (ABI-R) (Postmus et al., 2016b), the National Intimate Partner and Sexual Violence Survey (Thompson, 2006), the SVAWS (Marshall, 1992), the Scale of Economic Abuse-12 (Postmus et al., 2016a), the National Intimate Partner and Sexual Violence Survey (Thompson, 2006), the Safety Rating Scale (Culbertson et al., 2001), and the Violence Against Women Survey (Macmillan et al., 2000)	To detect fatality risk indicators of IPF
Dutton et al. (2019)	USA	168 police officers and 63 victim advocates of domestic violence agencies who administer the LAP	Modified version of officer survey and advocate survey (Maryland Network Against Domestic Violence)	To assess experiences and perceptions of police officers and victim advocates in the collaboration to apply the LAP
Ward-Lasher et al. (2018)	USA	266 police-involved victims of PVW	Official police records, interviews, the CTS-2 (Straus et al., 1996) and the DA (Campbell et al., 2003)	To examine police officers' decisions to make arrests in PVW/IPF cases based on violence and homicide risk assessment
Brignone and Gomez (2017)	USA	263 women patients of emergency departments (including victims of PVW)	The DA (Campbell et al., 2009)	To identify the women who visit emergency departments at highest risk of IPF
Messing and Campbell (2016)	USA	549-570 victims of PVW	The Lethality Screen (Messing et al., 2015a) and the DA-LE (Messing and Campbell, 2016)	To analyze the predictive validity of the Lethality Screen and the DA-LE
Messing et al. (2015b)	USA	1252 victims of PVW	Structured interviews, an adapted version of the safety-promoting behavior checklist (McFarlane et al., 2004), the CTS-2 (Straus et al., 1996), the DA (Campbell, 1986) and the Lethality Screen (Messing et al., 2015a)	To assess the effectiveness of the LAP used by police-social services on victim-survivors at risk of PVW/IPF to the adoption of safety strategies
Messing et al. (2014)	USA	432 women victims of PVW	Structured interviews, the DA (Campbell et al., 2003), the CTS2 (Straus et al., 1996), the women's experience of battering scale (Smith et al., 1995), and an adapted version of the safety-promoting behavior checklist (McFarlane et al., 2004)	To study the connection of homicide risk and safety actions among women victims of PVW
Messing et al. (2013)	USA	148 immigrant victims of PVW	Structured interviews, the CTS-2 (Straus et al., 1996), the DA (Campbell et al., 2009), the Women's Experience of Battering Scale (Smith et al., 1995) and the HARASS Scale (Sheridan, 1998)	To adapt the DA to immigrant women population
Messing et al. (2020a)	USA	1008 women victims of PVW	Structured interview, the DA (Campbell et al., 2003, 2009), and the Danger Assessment for Immigrants (DA-I) (Messing et al., 2013)	To examine the relationship between strangulation, loss of consciousness due to strangulation, and risk of future near-fatal violence to modify the DA and the DA-I
Bianchi et al. (2014)	USA	300 victims of PVW	The DA (Campbell, 1986) and the SAVAWS (Marshall, 1992)	To describe the demographics, frequency, severity of abuse, and the risk of murder for women who are abused during pregnancy in comparison with non-pregnant women
Campbell (1986)	USA	79 victims of PVW	Interviews, The CT (Straus, 1979) and the DA (Campbell, 1986)	To develop the DA to assess the danger of IPF and describing the literature supporting it
Anderson et al. (2021)	USA	88 male offenders of PVW/IPF (37 monitoring offenders and 51 non-monitoring offenders)	Official data from Domestic Violence High-risk Team Monitoring (DVHRT) and the LAP (Maryland Network Against Domestic Violence)	To analyze the association between the LAP and DVHRT and prosecution and sentencing outcomes of PVW/IPF offenders
Storey and Hart (2014)	Canada	100 cases of PVW	File review of the cases from the British Columbia Courts Services database and using the Spousal Assault Risk Assessment Guide (SARA) (Kropp, 1994), the DA (Campbell et al., 2009), the Ontario Domestic Assault Risk Assessment (ODARA) (Hilton et al., 2004), the Brief Spousal Assault Form for the Evaluation of Risk (B-SAFER) (Kropp et al., 2005)	To assess the validity of the DA
López-Ossorio et al. (2021)	Spain	2159 cases of PVW/IPF (2000 cases of PVW and 159 cases of IPF)	Official data from the VioGén System which collect and manage national information of intimate partner violence against women cases	To develop and validate a new scale to improve intimate partner homicide prediction
Nesset et al. (2017)	Norway	124 cases of PVW	Police reports data on emergency visits in cases of PVW/IPF and a Norwegian translation of the original Swedish version of the B-SAFER (Kropp et al., 2011)	To appraise the associations between of risk assessment and immediate protective actions by police as arrest and relocation of victims
Cunha and Goalves (2016)	Portugal	172 male aggressors (137 of PVW and 34 of IPF)	The SARA (Kropp et al., 1999)	To explore the differences between PVW and IPF and to identify the specific variables that predict IPF
Messing et al. (2017)	USA	1081 victims of PVW and attempted IPF	Structured interviews, the CTS-2 (Straus et al., 1996) and the Danger Assessment-5 (DA-5) (Snider et al., 2009)	To assess the predictive validity of the DA-5 adding a strangulation item to estimate the risk of attempted IPF
Wang (2015)	China	543 victims of PVW and attempted IPF	The Lethal Assault Checklist and the Taiwan Intimate Partner Violence Danger Assessment (TIPVDA) (Wang, 2012)	To evaluate the predictive validity of the TIPVDA to predict IPF
Glass et al. (2010)	USA	90 women victims of PVW	Structured interviews, the Decisional Conflict Scale (DSC) (O'Connor, 1995), the DA (Campbell et al., 2009)	To create and test a computerized safety decision aid for setting a protection plan to the risk for PVW
Campbell et al. (2009)	USA	310 IPF, 194 attempted IPF, 324 PVW cases	Structured interviews and the DA (Campbell, 1986; Campbell et al., 2003)	To develop and validate a weighted scoring for the DA-revised
Williams et al. (2021)	USA	4,665 men aggressors of PVW	The Domestic Violence Screening Instrument-Revised (DVSI-R) (Williams and Grant, 2006) and the DA (Campbell et al., 2009)	To determine the validity of a dual assessment protocol for persistence and potential lethality in PVW
Snider et al. (2009)	USA	666 victims of PVW of whom 400 completed follow-up interviews	Structured interviews and the DA (Campbell et al., 2009)	To design a risk assessment of severe violence or IPF for healthcare settings
Echeburúa et al. (2009)	Spain	269 men aggressors of IPF and attempted IPF, and 812 cases of PVW	Interviews and the Severe Intimate Violence Partner Prediction Scale (SIVIPAS) (Echeburúa et al., 2009)	To develop a scale that predict IPF and attempted IPF

#### 3.2.1. Country and Year

There are differences in dates and countries of the studies. The first study was published in 1986 in the United States (Campbell, 1986), and it has continued similarly in subsequent years. Twenty-seven studies were located in the United States to date, in each of the following years: Campbell (1986), McFarlane et al. (1998), Glass et al. (2008), Campbell et al. (2009); Glass et al. (2009); Snider et al. (2009), Glass et al. (2010), Messing et al. (2013), Bianchi et al. (2014); Messing et al. (2014), Messing et al. (2015a,b), Messing and Campbell (2016); Messing et al. (2016), Brignone and Gomez (2017); Grant and Cross-Denny (2017); Messing et al. (2017), Dutton et al. (2018); Ward-Lasher et al. (2018), Dutton et al. (2019); Richards et al. (2019); Sabri et al. (2019), Johnson et al. (2020); Messing et al. (2020a,b) and Anderson et al. (2021); Williams et al. (2021). Other studies have been done, for the last years, in other countries such as Spain in and Echeburúa et al. (2009), Canada in Storey and Hart (2014), China in Wang (2015), Portugal in Cunha and Goalves (2016), and Norway in Nesset et al. (2017), Spain in López-Ossorio et al. (2021) and Echeburúa et al. (2009).

#### 3.2.2. Sample

The sample size varied among the studies between 16 and 4,665 participants. This is constituted by a common sample of women victims of IPF, attempted IPF, and PVW by current or former male partners in heterosexual relationships (Campbell, 1986; McFarlane et al., 1998; Glass et al., 2008, 2009, 2010; Campbell et al., 2009; Snider et al., 2009; Messing et al., 2013, 2014, 2015a,b, 2016, 2017, 2020a,b; Bianchi et al., 2014; Storey and Hart, 2014; Wang, 2015; Messing and Campbell, 2016; Brignone and Gomez, 2017; Nesset et al., 2017; Richards et al., 2019; Sabri et al., 2019; Johnson et al., 2020; López-Ossorio et al., 2021). These aggressors are also included (Echeburúa et al., 2009; Storey and Hart, 2014; Cunha and Goalves, 2016; Nesset et al., 2017; Anderson et al., 2021; López-Ossorio et al., 2021; Williams et al., 2021). In some studies, professionals involved with the victims as advocates and police officers are considered sample as well (Grant and Cross-Denny, 2017; Dutton et al., 2018, 2019; Ward-Lasher et al., 2018).

#### 3.2.3. Data Sources

Most of the studies used similar data collection strategies from interviews and questionnaires directly from victims (Campbell, 1986; McFarlane et al., 1998; Glass et al., 2008, 2009, 2010; Campbell et al., 2009; Snider et al., 2009; Messing et al., 2013, 2014, 2015b, 2016, 2017, 2020a; Bianchi et al., 2014; Wang, 2015; Brignone and Gomez, 2017; Richards et al., 2019; Sabri et al., 2019; Johnson et al., 2020), aggressors (Snider et al., 2009; Messing et al., 2015a; Cunha and Goalves, 2016; Williams et al., 2021) and professionals (Dutton et al., 2018, 2019; Ward-Lasher et al., 2018). One uses focus groups (Grant and Cross-Denny, 2017). Secondary data were also extracted from official records and reports of legal and police databases (Storey and Hart, 2014; Nesset et al., 2017; Ward-Lasher et al., 2018; Anderson et al., 2021; López-Ossorio et al., 2021).

#### 3.2.4. Purpose of the Studies

The aims of the studies were diverse in content. The majority are focused on developing risk assessment instruments for IPF as well as analyzing their validity (Campbell, 1986; Campbell et al., 2009; Echeburúa et al., 2009; Snider et al., 2009; Messing et al., 2013, 2015a, 2017, 2020a; Storey and Hart, 2014; Wang, 2015; Messing and Campbell, 2016; López-Ossorio et al., 2021). There are several that assess the implications of them into professional practice (Glass et al., 2010; Messing et al., 2014, 2015b, 2016; Grant and Cross-Denny, 2017; Nesset et al., 2017; Dutton et al., 2018, 2019; Ward-Lasher et al., 2018; Richards et al., 2019; Sabri et al., 2019; Anderson et al., 2021; Williams et al., 2021). A few examine the risk factors of IPF using the existing risk assessment instruments (McFarlane et al., 1998; Glass et al., 2008, 2009; Bianchi et al., 2014; Cunha and Goalves, 2016; Brignone and Gomez, 2017; Johnson et al., 2020).

### 3.3. Main Findings

**Figure 2:**
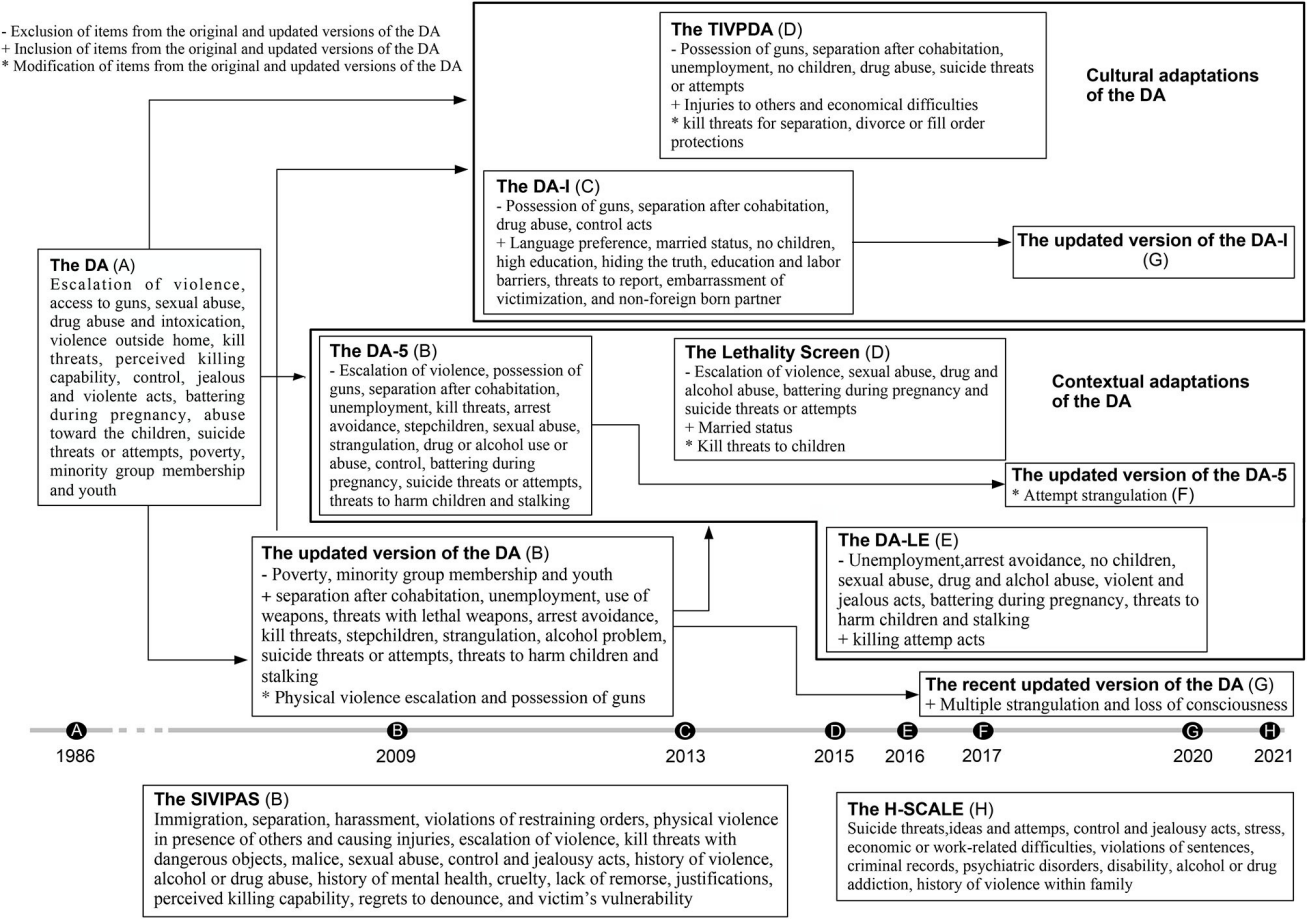
Evolution of the instrument's risk factors items.

The risk assessment instruments for IPF detected are Danger Assessment (DA), Danger Assessment for Immigrants (DA-I), Danger Assessment for Law Enforcement (DA-LE), Danger Assessment-5 (DA-5), the Taiwan Intimate Partner Violence Danger Assessment (TIPVDA), the Severe Intimate Partner Risk Prediction Scale (SIVIPAS), the Lethality Screen, and the H-Scale. Table 2 contains descriptions and psychometric properties related to the reliability and validity of these instruments. Figure 2 provides an overview of the number and content of the risk factors of said instruments.

**Table 2:** Risk asssessment instruments for IPF.

**Instruments**	**References**	**Definition**	**Items**	**Outcome assessed**	**Reliability**	**Validity**
The original DA	Campbell, 1986	It is an instrument that assess the danger of homicide in women by their intimate current or former partner	15	Attempted IPF	Cronbach's α = 0.71	Construct validity = significant correlation between DA and related constructs of severity-weighted index (r = 0.55; Probability[P] = 0.000), severity of worst injury (r = 0.50; P = 0.000) and severity of violent tact used against woman (r = 0.43; P = 0.000)
The updated version of DA	Campbell et al., 2009; Storey and Hart, 2014	It is an adapted version of the DA including additional risk factors of homicide against women by intimate partners	20	IPF and attempted IPF	Intraclass correlation coefficient = 0.83	AUC = 0.916(p <0.001; 95% Confidence Interval[CI]0.892 to 0.941). Sensitivity = 0.79. Specificity = 0.86/ AUC for a specific subset who had previously interfaced with a criminal justice, health care or victims' service agency = 0.862(p <0.001; 95% CI 0.812 to 0.913). Sensitivity for the mentioned subset = 0.82. Specificity for the mentioned subset = 0.76
The recent updated version of DA	Messing et al., 2020a	It is an updated version of the DA including an additional risk factor of homicide against women by intimate partners	20	Attempted IPF	Not reported	AUC = 0.70 (95% CI 0.638 to 0.751)^1^ and 0.71 (95% CI 0.638 to 0.774)^2^. Sensitivity = 0.69^1^ and 0.75^2^. Specificity = 0.56^1^ and 0.62^1^. PPV = 0.20^1^ and 0.60^2^. NPV = 0.89^1^ and 0.93^2^
The DA-I	Messing et al., 2013	It is a version of the DA adapted to immigrant women population	26	Any IPW and severe violence including attempted IPF	Not reported	AUC of severe violence = 0.85. AUC of any violence = 0.78
The updated version of the DA-I	Messing et al., 2020a	It is an updated version of the DA including an additional risk factor of homicide against immigrant women by intimate partners	Not reported clearly	Attempted IPF	Not reported	AUC = 0.838 (95% CI 0.748 to 0.928). Sensitivity = 0.86. Specificity = 0.63. PPV = 0.28. NPV = 0.96
The DA-LE	Messing and Campbell, 2016; Messing et al., 2020b	It is a version of the DA adapted to law enforcement context	11	Attempted IPF	Cronbach's α 0.75^3^-0.76^4^	AUC = 0.69 (95% CI 0.6139–0.7590)^3^. AUC = 0.75 (95% CI = 0.6785–0.8246)^4^. Sensitivity = 0.53^3^ and 0.65^4^. Specificity = 0.72^3^ and 0.77^4^. PPV = 0.16^3^ and 0.28^4^. NPV = 0.94^3^ ^4^
The DA-5	Snider et al., 2009	It is a brief version of the DA adapted to healthcare settings	5	Attempted IPF	Not reported	AUC = 0.79 (95% CI 0.73 to 0.85). Sensitivity = 0.83. Specificity = 0.56. PPV = 0.25. NPV = 0.95
The updated version of DA-5	Messing et al., 2017	It is an updated version of the DA-5 modifying risk items	5	Attempted IPF	Not reported	AUC = 0.69 (95% CI = 0.63 to 0.75). Sensitivity = 0.74, Specificity = 0.53, PPV = 0.19, NPV = 0.93
The TIPVDA	Wang, 2015	It is a version of the DA adapted to Chinese context	15	Attempted IPF	Cronbachs α = 0.73 - 0.77	AUC for predict both current and past lethal assault = 0.86. AUC for predict current lethal assault with no past = 0.72. AUC for predict past lethal assault with no current = 0.80. AUC for predict both current and past lethal assault, current lethal assault with no past, and past lethal assault with no current = 0.78
The SIVIPAS	Echeburúa et al., 2009	It is an instrument that identifying women victims of PVW who are at risk for attempted homicide and homicide by their intimate current or former partner	20	IPF and attempted IPF	Cronbachs α = 0.71	Sensitivity = 0.48. Specificity = 0.81
The Lethality Screen	Messing et al., 2015a; Messing and Campbell, 2016	It is an adaptation of the DA developed for first responders to predict severe violence and homicide in PVW cases	11	Near fatal violence (attemped IPF), severe violence, any PVW, and abuse	Not reported	Sensitivity for near fatal violence = 0.93. Specificity for near fatal violence = 0.21. PPV for near fatal violence = 0.13. NPV for near fatal violence = 0.96 / Sensitivity for severe violence = 0.93. Specificity for severe violence = 0.22. NPV for severe violence = 0.93. PPV for severe violence = 0.22 / Sensitivity for any PVW = 0.87. Specificity for any PVW = 0.22. NPV for any PVW = 0.80. PPV for any PVW = 0.32 / Sensitivity for abuse = 0.84. Specificity for abuse = 0.24. NPV for abuse = 0.48. PPV for abuse = 0.64 / Sensitivity = 0.57. Specificity = 0.56
The H-Scale	López-Ossorio et al., 2021	It is an instrument that estimate the risk of women homicide by their intimate current or former partner	13	IPF	Not reported	AUC = 0.81(95% IC 0.76 to 0.86)^5^. AUC = 0.80 (95% IC 0.74 to 0.86)^6^. Sensitivity = 0.81^5^ and 0.84^6^. Specificity = 0.61^5^ and 0.60^6^. PPV = 0.19 ^5, 6^. NPV = 0.97^5, 6^

*1: It is a subset used to develop an updated version of the DA*.

*2: It is a subset used to validate the updated version of the DA*.

*3: It is a subset used to develop the DA-LE*.

*4: It is a subset used to validate the DA-LE*.

*5: It is a subset used to develop the H-Scale*.

*6: It is a subset used to validate the H-Scale*.

#### 3.3.1. Brief Description

The DA (Campbell, 1986) is the first risk assessment instrument to assist women victims in estimating their danger of homicide or severe injury by current or former male partners. It was originally developed based on a review of scientific literature on risk factors for IPF and serious injuries from PVW, expert knowledge, and information from abused women (Campbell, 1986). Later studies examined the instrument with abused women, using updated versions (Campbell et al., 2009; Storey and Hart, 2014; Messing et al., 2020a). These women did not die, but most of them suffered near-fatal violence, so the outcome measure of the DA is not considered IPF but rather an attempted IPF. Only one study of the updated version also included IPF cases. The DA has been adapted to a culturally competent risk assessment instrument for abused immigrant women based on information from representative victims. This instrument is named DA-I (Messing et al., 2013) and its outcome measure is the prediction of any PVW and severe violence, including attempted IPF (Messing et al., 2013). A subsequent study updated this instrument, focusing on the prediction on attempted IPF (Messing et al., 2020a). The DA has also been adapted to abused Chinese women referred to TIPVDA (Wang, 2015), being its outcome measure attempted IPF too (Wang, 2015).

The DA has not only been adapted to specific populations but also different contexts such as law enforcement known as DA-LE (Messing and Campbell, 2016; Messing et al., 2020b). It is conceived to be used by professionals involved in PVW cases as domestic violence practitioners and police officers, as a risk-informative tool to identify high-risk PVW cases and intervene if needed. This instrument was developed using the information received from PVW battered women, some of whom have suffered near-lethal violence. Thus, the DA-LE has the outcome measure of predicting attempted IPF (Messing and Campbell, 2016; Messing et al., 2020b). The DA has been adapted to the healthcare area as well, being termed DA-5 (Snider et al., 2009). It is a brief risk assessment for acute care settings that intend to identify battering women at risk of severe injury or near-lethal violence by intimate partners. Thus, the outcome measure is to predict attempted IPF based on previous information compiled by battered women, including survivors of IPF. A later study updated this version centered on this outcome too (Messing et al., 2017). The Lethality Screen (Messing et al., 2015a; Messing and Campbell, 2016) is another adaptation of the DA for first responders that are involved in PVW cases as risk-informed collaborative interventions. It was created to identify high-risk victim-survivors. This was developed with battered women, including near-fatal violence and severe violence, but not homicide cases. Thus, its outcome measure is attempted IPF (Messing et al., 2015a; Messing and Campbell, 2016). Additionally, there is a program named the Lethality Assessment Program (LAP) composed of the Lethality Screen and the Lethality Assessment Protocol. It not only allows detecting victims in danger but also connects them with professionals to conduct an intervention to prevent the fatal result (Messing et al., 2015a; Messing and Campbell, 2016).

There are instruments independent of the DA that are SIVIPAS (Echeburúa et al., 2009) and H-Scale (López-Ossorio et al., 2021). Both are scales and have as measure the prediction of IPF in distinction with non-serious and non-lethal violence. SIVIPAS is also sensible for attempted IPF. Thus, these are used by the police, judicial, and social services professionals (Echeburúa et al., 2009; López-Ossorio et al., 2021).

#### 3.3.2. Number and Content of Risk Factor Items

The risk factors items of the instruments have been changing in number and content over time. It is presented in Figure 2 and elaborated in the following paragraphs.

The original version of the DA (Campbell, 1986) has 15 risk factors of IPF. These include: escalation of frequency and severity of violence; presence of armed guns in the house; sexual abuse; batterer abuses drugs and/or daily intoxication; violent outside the house; death threats or her belief in that he is capable of it; controlling all aspects of her life; violent jealousy; physical violence during pregnancy; abuses toward the couple's children; suicide threats or attempts by the victim; economic incomes below the poverty line; minority group membership; and women age between 15 and 34 years (Campbell, 1986). The updated versions of DA (Campbell et al., 2009; Storey and Hart, 2014; Messing et al., 2020a) contain 20 items, being the last three items not included. Other modifications were introduced as a specification of the physical violence escalation; and guns in the house was replaced for aggressor possesses his own guns. Other items were added, including: she left him after living together; he is unemployed; he uses weapons against her; he threatened her with a lethal weapon; he avoided being arrested for domestic violence; the woman has children that are not his; he tried to strangle or choke her multiple times resulting in loss of her consciousness; he is an alcoholic or problematic drinker; he threatened or tried to commit suicide; he threatened her to harm her children; and he stalked her (Campbell et al., 2009; Storey and Hart, 2014; Messing et al., 2020a). The instrument incorporates four levels of danger depending on how many items are checked out to be true. These are variable danger (less than 8 items), increased danger (8–13), severe danger (14–17), and extreme danger (18 and above) (Campbell et al., 2009; Storey and Hart, 2014).

The DA-I (Messing et al., 2013) has 26 items, most of them from the DA. Those not included in the DA are: possessing own guns; she left him after living together; battered use of drugs; and he controls all aspects of her life. Additional risk factors specific to immigrant women were added, such as her language preference to answer questions; she is married to the aggressor; they have children in common or not; she has college, vocational school, and/or graduate school degrees; she hides the truth from others because of fear of him; he obstacles her school attendance, getting job training or learning English; he threatened to report her to Child Protective Services, immigration, or other authorities; she feels ashamed of things he does; and he was born in the United States. These items have different weights. Death threats; violently jealous aggressor; no kids in-home; no common children; and victim shaming have the highest weights. The lowest weight items are: she believes that he is capable of killing her; battering during pregnancy; aggressor suicide threats or attempts; avoidance of being arrested for domestic violence; stalking; the language of the interview; victims hide the truth from others; and high education (Messing et al., 2013). The updated version of the DA-I has no substantial modifications (Messing et al., 2020a). The four levels of danger are maintained except for changes on the scores included in each: variable danger (less than 14), increased danger (15–25), severe danger (26–35), and extreme danger (36 and above) (Messing et al., 2013).

The TIPVDA (Wang, 2015) has 15 items eliminating some included in the DA, which are aggressor possess own guns; women left him after living together; unemployment; non-common children; use of drugs; and aggressor and victim suicide threats or attempts. This instrument includes new threats to women if she separated, divorced, sought professional help, or filed an order of protection; aggressor physically hurt other people; and experiencing financial stress or difficulties. More than eight affirmative responses to items of 15 are considered high-risk (Wang, 2015). The DA-LE (Messing and Campbell, 2016; Messing et al., 2020b) includes 11 items from the DA, but removing unemployment; avoidance of being arrested for domestic violence; non-common children; sexual abuse; use of drugs; alcohol or problematic drinker; violent and constant jealousy; battering during pregnancy; threats to harm children; and stalking. The item batterer tried to kill woman has also been integrated into the tool. Each affirmative answer to the items has been assigned one point, establishing this instrument high-risk cases above a score of 7 out of 11 (Messing and Campbell, 2016; Messing et al., 2020b).

The Lethality Screen (Messing et al., 2015a; Messing and Campbell, 2016) is also integrated by 11 items from DA with some modifications. The item threats to hurt children is replaced for threats to kill them. One new item is if she is married to him. Deleted items are: escalation of violence; sexual abuse; use of drugs, alcoholic or problematic drinker; battering during pregnancy; and women with suicide threats or attempts. It establishes two levels of risk based on the affirmative responses: not serious danger (less than 7) and high danger (7 and above) (Messing et al., 2015a; Messing and Campbell, 2016). The DA-5 (Snider et al., 2009) is another brief version of DA with a total of 5 items. The items are from DA, but only include the escalation of physical violence in frequency and severity; use of weapons; her believing that he is capable of killing her; battering during pregnancy; and violent jealousy, excluding the rest (Snider et al., 2009). The updated version of the DA-5 introduces attempted strangulation in replacement for battering during pregnancy (Messing et al., 2017). The presence of at least 3 out of 5 affirmative answers is considered high-risk (Snider et al., 2009; Messing et al., 2017).

The SIVIPAS (Echeburúa et al., 2009)is not based on the items from DA, integrating 20 items. Some of them are similar to the included in the DA as separation; harassment; escalation of violence; severe or kill threats; threats with dangerous objects or weapons; sexual abuse; intense jealousy or controlling behaviors; abuse of alcohol and/or drugs; and victim perception of the danger of death. The specific items included are man or woman immigrant; breaking restraining orders; physical violence in the presence of others and that cause injury; intention of causing severe injuries; history of violence against other people including previous partners; man with mental illness and dropping out of psychiatric or psychological treatments; cruel; disparaging behaviors; lack of remorse; justification of violent behavior; victim attempts to drop charges or goes back on her decision to either leave or report the aggressor to the police; victim's vulnerability because of illness; solitude or dependence. It defines three levels of risk depending on the affirmative responses to items: low (less than 4), moderate (5–9), and high (10 and above) (Echeburúa et al., 2009). The H-Scale (López-Ossorio et al., 2021) is another instrument developed independently from the DA. It has 13 items, including victim and aggressor suicide threats and attempts; exaggerated jealousy and controlling; and economic or work-related problems similar to the included on the DA. Problems in aggressor life stressful; physical or sexual aggression records; past breakings of sentence conditions; victim and aggressor mental or psychiatric disorder; victim with any disability or addiction in drugs or alcohol; and aggressor history of gender or domestic violence within victim's family. It has five levels of risk based on the presence or absence of items: unappreciated, low, medium, high, and extreme (López-Ossorio et al., 2021).

#### 3.3.3. Reliability and Validity

Only five studies focused on the reliability of the instruments, assessing four of the internal consistency reliability using Cronbach's α and one interrater reliability with intraclass correlation. These studies reported a Cronbach's α of 0.71 for the original DA (Campbell, 1986), 0.75–0.76 for the DA-LE (Messing and Campbell, 2016; Messing et al., 2020b), 0.73–0.77 for TIPVDA (Wang, 2015), and 0.71 for SIVIPAS (Echeburúa et al., 2009); and an intraclass correlation coefficient of 0.83 for the updated version of the DA (Storey and Hart, 2014). All studies examine criterion-related validity through predictive validity, including AUC-ROC, sensitivity, specificity, PPV, and NPV, except one study that exclusively assesses construct validity. This measure was issued for the original DA revealing a significant correlation between this instrument and construct of the severity-weighted index (*r* = 0.55), the severity of worst injury (*r* = 0.50), and severity of violent tact used against women (*r* = 0.43) (Campbell, 1986). The predictive validity of all instruments is not comparable due to the heterogeneity of the outcomes assessed, being analyzed by separated categories.

Firstly, the instruments that predict attempted IPF (Table 2) have an AUC ranging from 0.680 to 0.916. It included 0.695 to 0.706 for the recent updated version of DA (Messing et al., 2020a), 0.775–0.852 for the DA-I (Messing et al., 2013), 0.838 for the updated version for the DA-I (Messing et al., 2020a), 0.686–0.752 for the DA-LE (Messing and Campbell, 2016; Messing et al., 2020b), 0.79 for the DA-5 (Snider et al., 2009), 0.69 for the updated version of DA-5 (Messing et al., 2017), 0.718–0.856 for the TIPVDA (Wang, 2015). There is not information of the AUC for the Lethality Screen (Messing et al., 2015a; Messing and Campbell, 2016).

The sensitivity and specificity of these instruments are based on cut-point scores. The recently updated version of DA has a sensitivity ranging from 0.69 to 1, and a specificity from 0 to 0.62, being both more balanced in the extreme danger level (cutoff score of 18 and above) with a sensitivity of 0.69 and 0.75 (for training and test samples, respectively) and specificity of 0.56 and 0.62 (for training and test samples, respectively) (Messing et al., 2020a). Sensitivity and specificity data for the DA-I has not been identified (Messing et al., 2013), but there is data for the updated version of the DA-I: the sensitivity varies from 0.29 to 1, and specificity ranges from 0.97 to 0, being the most balanced in the severe danger level (cutoff score between 26 and 35) with a sensitivity of 0.86 and specificity of 0.63 (Messing et al., 2020a). The DA-LE has a sensitivity ranging from 0.39 to 1 and specificity from 0 to 1, being both maximized at the cutoff score of 7 with a sensitivity between 0.53 and 0.65 (for training and test samples, respectively) and a specificity between 0.72 and 0.77 (for training and test samples, respectively) (Messing and Campbell, 2016; Messing et al., 2020b). The sensitivity of the DA-5 ranged from 0.83 to 1 and the specificity from 0.15 to 0.56, being both maximized at the score 3 with a sensitivity of 0.83 and specificity of 0.56 (Snider et al., 2009). The updated version of the DA-5 has a sensitivity range from 0.25 to 0.96 and specificity from 0.13 to 0.92, being the most balanced in at the score 3 with a sensitivity of 0.74 and specificity of 0.53 (Messing et al., 2017). The sensitivity and specificity of the Lethality Screen are not clear due to studies showing different data. One of them reports a sensitivity of 0.93 and specificity of 0.21, not showing cut-points (Messing et al., 2015a). Another one reveals a cutoff score but not a point at which both be balanced, with a high sensitivity observed at low specificity, and vice versa. However, it is observed that the maximum equilibrium is at the score of 7 with a sensitivity of 0.57 and specificity of 0.56 (Messing and Campbell, 2016).

PPV and NPV were less common measures of predictive validity, being reported in a few instruments. For the recently updated version of DA, the PPV was varied from 0.13 to 0.60 and the NPV from 0 to 0.93, depending on the cutoff scores. For the extreme danger level score (balanced rating of sensitivity and specificity cutoff), the PPV was around 0.20 and 0.60 (for training and test samples, respectively), and the NPV of 0.89 and 0.93 (for training and test samples, respectively) (Messing et al., 2020a). The updated version of the DA-I has a different PPV and NPV based on the cut-points ranging the first from 0.14 and 0.60 and the second from 0 and 0.89. The most balanced sensitivity and specificity of this instrument were in the severe danger level, which has a PPV of 0.28 and NPV of 0.96 (Messing et al., 2020a). The DA-LE has a PPV ranging from 0.90 to 1 and a NPV from 0 to 0.93, being the sensitivity and specificity maximized at the cutoff score of 7, which PPV is between 0.16 and 0.28 (for training and test samples, respectively) and NPV is 0.94 (same for training and test samples) (Messing and Campbell, 2016; Messing et al., 2020b). The DA-5 has a PPV ranging from 0.17 to 0.57 and a NPV from 0.88 to 1, being the sensitivity and specificity maximized at the cutoff score of 3, which PPV is 0.25 and NPV is 0.95 (Snider et al., 2009). The updated version of DA-5 has a PPV ranging from 0.14 to 0.31 and a NPV from 0.89 to 0.96, being the sensitivity and specificity also maximized at the cutoff score of 3, which PPV is 0.19 and NPV is 0.93 (Messing et al., 2017). The Lethality Screen has a PPV of 0.13 and a NPV of 0.96 (Messing et al., 2015a; Messing and Campbell, 2016).

Secondly, for predicting both IPF and attempted IPF, there is the first updated version of the DA and the SIVIPAS. The updated version of DA has an AUC of 0.913-0.916. Its sensitivity ranged from 0.55 to 0.99 and the specificity from 0.53 to 0.97 across the DA danger level scores, being both maximized at the severe danger level (cutoff score between 14 and 17) with a sensitivity of 0.79 and specificity of 0.86. PPV and NPV have not been reported for this instrument (Campbell et al., 2009). SIVIPAS has a specificity ranging from 0 to 0.138 to 1 and a specificity from 0 to 1, being the ten cutoff score a sensitivity of 0.48 and specificity of 0.81. There are no AUC, PPV, or NPV data for this instrument (Echeburúa et al., 2009).

Thirdly, to predict IPF, the H-Scale is used, which has an AUC is 0.81 for the training sample and 0.80 for the test sample. The sensitivity is 0.81 for the training sample and 0.84 for the test sample, whereas the specificity is 0.61 for the training sample and 0.60 for the test sample. PPV is 0.19 and NPV is 0.97 (López-Ossorio et al., 2021).

Based on the instruments above' predictive capacity for IPF and attempted IPF, they are considered predictive models.

### 3.4. Quality Assessment Results

#### 3.4.1. Quality Results of the Search Strategy

The latent semantic analysis reveals that the combination of the keywords piloted in the databases allowed the identification of studies that respond to the research questions of the systematic review, being an indicator of the quality of the search strategy used. The mentioned analysis was carried out based on 26 studies of 33 due to the presence of keywords. These studies are grouped into three factors by associating the specific research themes included in the terms.

Factor 1 includes 16 studies that represent two thematic areas. First, 11 studies focused on analyzing risk factors for severe PVW and IPF and modifying some of them the existing risk assessment instruments according to the obtained fatality risk indicators. Second, five studies analyze the effect of applying risk assessment instruments and safety programs on victims' safety. The common theme of all studies included in factor 1 is the generation of scientific knowledge of the risk factors of severe PVW and IPF and the instruments and programs detecting and preventing it. The set of keywords of the 16 studies is semantically close, representing the mentioned common theme of factor 1. These keywords account for 61.54% of the variance attributable to the factor.

Factor 2 involves seven studies with two thematic areas. First, five studies test risk assessment instruments. Second, two studies examine the implementation of risk assessment and safety programs in daily professional practice. The common element of the studies included in factor 2 is the application of scientific knowledge to improve existing risk assessment instruments and protective programs. The group of keywords of the seven studies semantically close elements accounts for 26.92% of the variance attributable to the factor.

Factor 3 presents three studies with two thematic areas. First, two studies develop risk assessment instruments and safety programs adapted to specific areas and populations. Second, 1 study assesses the link between detecting lethal risk cases and the offender prosecution outcomes. The central theme of all the studies included in factor 3 is the expansion of knowledge and application in managing severe PVW and IPF. The set of keywords of the three studies for a total of 11.54% of the variance attributable to the factor.

The specific keywords identified in the studies refer to violence within intimate relationships, severe violence and homicide, and risk assessment instruments. The principal terms related to the violence within relationships used are “domestic violence” and “intimate partner violence”; related to severe violence homicide used are “homicide,” “lethality,” “lethal intimate partner violence,” “intimate partner homicide,” “intimate partner femicide,” “femicide,” and “spousal homicide.” Those related to risk assessment instruments of severe violence and homicide are “assessment,” “risk assessment,” “lethality assessment,” and “risk of murder.” These keywords are detected in the three factors and distinguished as the most explanatory of the variance in each factor. In particular, the most explanatory keywords of factor one are “intimate partner homicide,” “homicide,” “domestic violence,” “femicide,” “risk assessment”; factor two are “lethality assessment program,” “homicide,” “risk assessment,” “intimate partner homicide”; and of factor three are “intervention,” “lethality assessment program,” “intimate partner violence,” “domestic violence,” and “intimate partner homicide.”

The mentioned keywords referred to violence within relationships, severe violence, homicide, and risk assessment instruments are used in the three factors, but they do not equally account for the variance of each one. These terms account for a higher variance for factor one, acquiring more strength represented by citations. At the same time, the terms are less cited for the papers with topics included in factors two and three due to their lower variance. Additionally, there are specific keywords for each factor common among several papers within the same factor. This contributes to each factor representing a different theme. The main keywords that differentiate factor 1 from the rest are “risk factors” and “safety planning.” The main keyword differentiating factor 2 from the rest is “validity.” The main keywords differentiating factor 3 from the rest are “indigenous” and “legal intervention.”

#### 3.4.2. Quality Results of the Studies

On the quality of the 33 studies included in the systematic review, the Table 3 summarizes the quality scores of each one. The mean quality score is 21.03 of a possible 32. The standard deviation is 4.77 in the range of 14 and 31. These data indicate a medium-high quality. It reveals that the studies effectively determine objectives, participants recruitment, representative sample, outcomes, methods, measurement uniformity, and statistical tests. Most of the studies scored high in all elements of the selection bias, the first three elements of measurement bias, and the first element of reporting bias categories. The two elements of the reporting bias category have high scores, although not as marked as those above. This implies that the consideration of potential confounders and the generalization of results are adequate but not excellent. In contrast, most of the studies scored less on the last five elements of the measurement bias, the only element of the attrition bias, and the last three elements of the reporting bias categories. This means that the administration tool, use of multiple sources of information, follow-up, treatment of missing data, drop-out rates registration, and predictive validity record are acceptable.

**Table 3:** Quality scores of the studies included in the systematic review.

	**(A)**			**(B)**								**(C)**	**(D)**				**Score**

	**(a)**	**(b)**	**(c)**	**(d)**	**(e)**	**(f)**	**(g)**	**(h)**	**(i)**	**(j)**	**(k)**	**(l)**	**(m)**	**(n)**	**(o)**	**(p)**	
Messing et al. (2015a)	2	2	2	2	2	2	2	2	2	1	0	2	2	2	2	1	28
Messing et al. (2020b)	2	2	2	2	2	2	2	1	2	1	2	2	2	2	1	1	28
Richards et al. (2019)	2	2	2	2	2	2	0	0	2	1	0	2	2	0	2	2	23
Sabri et al. (2019)	2	2	2	2	2	2	0	0	2	2	0	1	2	0	2	2	23
Glass et al. (2009)	2	2	2	2	2	2	0	0	0	0	0	0	2	0	1	2	17
Glass et al. (2008)	2	2	1	2	2	2	0	0	0	0	0	0	2	0	1	1	15
McFarlane et al. (1998)	2	2	2	2	2	2	0	0	0	0	0	0	2	0	1	2	17
Dutton et al. (2018)	2	2	1	2	2	2	0	0	0	0	0	0	2	0	2	1	16
Grant and Cross-Denny (2017)	2	2	1	2	2	2	0	0	0	0	0	0	2	0	2	1	16
Messing et al. (2016)	2	2	2	2	2	2	0	0	0	0	1	0	2	0	0	2	17
Johnson et al. (2020)	2	2	2	2	2	2	0	0	0	0	1	0	2	0	1	2	18
Dutton et al. (2019)	2	2	2	2	2	2	0	0	0	0	2	0	1	0	0	2	17
Ward-Lasher et al. (2018)	2	2	2	2	2	2	0	0	2	2	0	2	2	0	1	2	23
Brignone and Gomez (2017)	2	2	2	2	1	2	0	0	0	0	2	0	0	0	0	2	15
Messing and Campbell (2016)	2	2	2	2	1	2	2	1	2	1	1	1	2	2	1	1	25
Messing et al. (2015b)	2	2	2	2	2	2	0	0	2	1	2	2	2	0	1	2	24
Messing et al. (2014)	2	2	2	2	2	2	0	0	0	0	2	0	2	0	1	2	19
Messing et al. (2013)	2	2	2	2	2	2	2	2	2	2	2	2	2	2	2	1	31
Messing et al. (2020a)	2	2	2	2	2	2	2	1	2	2	2	2	2	2	2	1	30
Bianchi et al. (2014)	2	2	2	2	2	2	0	0	2	2	0	0	2	0	0	2	20
Campbell (1986)	2	2	1	2	2	2	2	1	0	0	0	0	1	0	2	1	18
Anderson et al. (2021)	2	2	1	2	2	2	0	0	0	0	2	0	2	0	2	2	19
Storey and Hart (2014)	2	2	1	2	2	2	2	2	1	2	1	0	2	2	2	1	26
López-Ossorio et al. (2021)	2	2	2	2	2	2	2	1	0	0	0	0	2	2	2	2	23
Nesset et al. (2017)	2	2	2	2	2	2	0	0	0	0	0	0	2	0	2	2	18
Cunha and Goalves (2016)	2	2	2	2	2	2	0	0	0	0	0	0	2	0	1	2	17
Messing et al. (2017)	2	2	2	2	2	2	2	1	2	2	1	2	2	2	1	1	28
Wang (2015)	2	2	2	2	2	2	2	1	0	0	0	0	2	0	2	1	20
Glass et al. (2010)	2	2	1	2	2	2	0	0	0	0	0	0	1	0	1	1	14
Campbell et al. (2009)	2	2	2	2	2	2	2	1	0	0	0	0	2	2	1	2	20
Williams et al. (2021)	2	2	2	2	2	2	0	0	1	1	0	0	2	0	1	2	19
Snider et al. (2009)	2	2	2	2	2	2	2	0	2	2	0	2	2	2	2	1	27
Echeburúa et al. (2009)	2	2	2	2	2	2	2	1	0	0	0	0	2	2	2	2	23

*(A) Selection bias; (B) Measurement bias; (C) Attrition bias; (D) Reporting bias*.

*(a) Clear objectives*.

*(b) Participants were recruited in an acceptable way*.

*(c) Representative sample*.

*(d) Clear definition of outcome*.

*(e) Clear description of the methods*.

*(f) The outcome was measured in the same way across all participants*.

*(g) The tool was administered by professionals*.

*(h) Authors use multiple sources of information to score risk assessments*.

*(i) The follow-up period was sufficiently described and reported*.

*(j) The follow-up period was long enough*.

*(k) Missing data treatment*.

*(l) Drop-out rates were recorded; (m) Appropiate statistical tests*.

*(n) The predictive validity of the tests was reported*.

*(o) Potential confounders were taken into account*.

*(p) Generilizable results*.

## 4. Discussion

The present systematic review established three research questions to synthesize the scientific knowledge of risk assessment instruments for IPF: What are the specific risk assessments instruments for IPF?, What are the risk factors for IPF included in the instruments? And what are the reliability and validity of the instruments? The findings respond to them and are discussed in the following paragraphs.

Addressing the first research question, the instruments for IPF are identified in different studies included in the systematic review. The quality search strategy allowed us to obtain fundamental studies to comprehensively understand these instruments. It is an innovative aspect in this review since no quality assessment of the strategy has been identified in other studies in the area. In the study selection process, a high number of them didn't meet the inclusion criteria and were excluded. In the field of risk assessment, it is not common to use specific keywords to refer to violence against women by current or former male partners, so we used general terms related to intimate partner violence and homicide across the different databases, proceeding from general partner violence to particular partner violence against a specific gender. In some studies included, different terms were detected to refer to PVW and IPF, such as “intimate partner violence against women” and “intimate partner femicide.” The greater integration of these terms in the studies on the matter is essential to facilitate the identification of these studies and promote more visibility of violence against women. In addition, the quality assessment of the included studies allows the analysis of the risk of bias, not being an judgment on how good or bad they are.

Regarding study characteristics results, research on risk assessment instruments for IPF over time with updates and cultural and contextual adaptations is observed. However, most of the studies have been carried out in the United States, making it difficult to extrapolate the findings equally to all parts of the world, hence the need for further research in other countries. In addition, the analysis of the studies' characteristics has also allowed identifying samples based mainly on victims' self-reports which are not officially corroborated. This information is crucial, but focusing exclusively on the victim is insufficient. A completion with other sources is essential as evidence reveals that there are victims with distorted perceptions of victimization (Patró Hernández et al., 2011; Storey and Hart, 2014). Analysis of official data is also required to include lethal cases to contribute to a greater number of risk assessment instruments for IPF and not just for attempted IPF, which are the majority. Regarding the sample, some of the included studies use a recurrent one. These studies used sample data collected for the National Institute of Justice-funded Oklahoma Lethality Assessment Study (OKLA) and Risk Assessment Validation study (RAVE) (Messing et al., 2013, 2015a, 2017, 2020a,b; Messing and Campbell, 2016). This data was used to develop and test different risk assessment instruments for IPF, which poses limitations regarding the obtained solid results, highlighting the need to carry out future studies to validate or refute them using diverse samples.

The findings also respond to the second research question "what are the risk factors for IPF included in the instruments?". The most frequent factors identified in the instruments are validated by several studies that demonstrated an association of them with the IPF. It reveals that they are essential to continue to be part of the instrument to predict the phenomenon. These are escalation of frequency and severity of violence (Nicolaidis et al., 2003; Kivivuori and Lehti, 2012; Vatnar and Bjørkly, 2013; Cunha and Goncalves, 2016; Johnson et al., 2020; Monckton Smith, 2020), sexual abuse (Bagwell-Gray, 2016; Dobash and Dobash, 2016), victim's perception that aggressor is capable of killing her (Nicolaidis et al., 2003; Vatnar and Bjørkly, 2013; Johnson et al., 2020), drug and alcohol problems (Belfrage and Rying, 2004; Kivivuori and Lehti, 2012; Cunha and Goalves, 2016; Dobash and Dobash, 2016; Johnson et al., 2020), battering during pregnancy (Decker et al., 2004), suicide threats or attempts (Belfrage and Rying, 2004), separation (Belfrage and Rying, 2004; Dobash and Dobash, 2011; Cunha and Goncalves, 2016; Abrunhosa et al., 2021), kill threats (Nicolaidis et al., 2003; Belfrage and Rying, 2004; Cunha and Goncalves, 2016), stalking (Nicolaidis et al., 2003; Johnson et al., 2020), aggressor control daily victim's activities (Decker et al., 2004; Dobash and Dobash, 2011; Bagwell-Gray, 2016; Monckton Smith, 2020), aggressor is violently and constantly jealous of victim (Nicolaidis et al., 2003; Dobash and Dobash, 2011; Bagwell-Gray, 2016; Johnson et al., 2020), and access, possession and use of weapons (Cunha and Goncalves, 2016; Reckdenwald et al., 2019; Johnson et al., 2020; Monckton Smith, 2020; Abrunhosa et al., 2021).

There are more factors supported by numerous studies that show their association with IPF, but they are included in a small number of instruments, contrary to the previous case. These are low economic income, unemployment, young women, stepchildren, no children, marital status, history of violence, criminal records, violence with injuries, history of mental health problems, lack of remorse, justifications, minority membership, immigration, non-foreign born aggressor, and non-lethal threats. Concerning the factor of low economic income, several studies reveal that it is an element associated with the IPF. This could explain why other elements already included in the instruments are risk factors as unemployment without economic benefits nor pension (Fernández-Montalvo and Echeburúa, 2005; Cunha and Goncalves, 2016). Furthermore, the evidence specifies that the lack of work by itself also has an impact on IPF perpetration due to being retired or responsible for the household chores are also associated with the deaths that should be considered for inclusion in the instruments (Kivivuori and Lehti, 2012; Sebire, 2017; Ward-Lasher et al., 2018). The factor women age between 15 and 34 years the evidence clarifies the range itself is not relevant, but the fact that the victim is younger than the aggressor (Cunha and Goalves, 2016; Sebire, 2017). Thus, modifying this item to this condition should be considered. The presence of children who are not biological offspring of the aggressor is also corroborated by scientific studies but has not yet been identified. This association with childlessness requires more research and needs to be explored (Sebire, 2017; Soria Verde et al., 2019). Regarding the marital status, it is a factor associated with the IPF. Also, serious partnerships without this status that are living together is another element to be considered for inclusion on the instruments (Dobash and Dobash, 2016). The evidence points at the history of interpersonal violence as a strong predictor of IPF. It could be evidenced in criminal records, and both are integrated into only a few instruments (Kivivuori and Lehti, 2012; Dobash and Dobash, 2016; Sebire, 2017; Soria Verde et al., 2019; Monckton Smith, 2020).

The use of violence resulting in injuries is a significant risk indicator to IPF when it is on the victim's face, head, or neck, which is not specified in the instruments (Reckdenwald et al., 2019). The factor history of mental health problems could be complete with the evidence, indicating the studies that the presence of any mental disorder in the aggressor is not associated with IPF. Specifically, anxiety, affective, psychotic, and personality disorders are related to it (Belfrage and Rying, 2004; Cunha and Goalves, 2016; Caman et al., 2022). Moreover, the suicide threats or attempts risk factor items commonly included in the tools are a manifestation of the affective disorder and personality disorder such as depressive disorder and borderline personality disorder (Belfrage and Rying, 2004; Caman et al., 2022). The above elements of suicide are related to the victim's danger of death, but also that of the aggressor as evidence indicates that a significant proportion of IPF cases are followed by the suicide of the offender (Vatnar et al., 2022). Simultaneous lack of empathy and remorse are factors associated with IPF (Dobash and Dobash, 2011, 2016). Although not included in the instruments, this evidence suggests the need for consideration in the future. Cognitive justifications to violence by aggressors are an important factor associated with IPF because they neutralize these acts (Fernández-Montalvo and Echeburúa, 2005; Dobash and Dobash, 2011). Immigration is an evidenced factor associated with IPF, and it could be interconnected with the other non-foreign born aggressor factor due to the link to lethal violence is greater when victim and aggressor are immigrants and come from the same ethnic background (Belfrage and Rying, 2004). Acculturation stress, welfare deficiencies and ethnic discrimination could be related to the involvement of immigrants with IPF (Vatnar et al., 2017). Ethnic minority membership is another factor related to immigration validated by scientific studies (Belfrage and Rying, 2004; Fernández-Montalvo and Echeburúa, 2005; Cunha and Goncalves, 2016; Sebire, 2017; Ward-Lasher et al., 2018). Death threats is another factor commonly repeated in the instruments, but it is not for threats against the victim to harm children and report to Child Protective Services, immigration, and other authorities. This is in the face of the victim's desire to denounce the victimization, seeks professional help, or separation/divorce, which are also included in the instruments with low frequency. More research is needed to validate or refute this inclusion to discriminate between lethal and non-lethal violence.

Evidenced factors associated with IPF and not included in the instrument have been identified. Specifically, regarding the aggressor these are protection order and prison sentences (Kivivuori and Lehti, 2012; Dobash and Dobash, 2016; Monckton Smith, 2020), childhood problems (Dobash and Dobash, 2016), distorted beliefs and rigid cognition of aggressor about possessiveness and control over women and fear of abandonment (Nicolaidis et al., 2003; Dobash and Dobash, 2016; Monckton Smith, 2020). Relative to victims there are isolation and submissive behaviors factors (Sebire, 2017; Monckton Smith, 2020). It would be convenient to consider incorporating these factors in the instruments and examining the improvement of psychometric properties with this inclusion. Nevertheless, not only the content and number of factors should be considered, but also the combination of these together to predict IPF. The study of Dawson and Piscitelli (2021) identified that the relationship between the number of factors and the risk of death is not necessarily linear. Different clusters of homicide have been defined and each of them has unique combinations of risk factors. The study of Gnisci and Pace (2016) reveals that IPF is a dynamic process and the sequence in which risk factors appear is important as well as the number and grouping of them. Some elements may be predictive of IPF by themselves, but when combined sequentially with others that occur, their predictive strength may be greater. Therefore, this heterogeneity must be taken into account for the prediction and prevention. Future studies could analyze in representative groups of PPW and IPF a wide variety of factors (related to victim, aggressor, relationship and environment) and consider how the combination of these and their occurrence over time increase or decrease the likelihood of homicide.In other words, not to focus attention on identifying specific factors are risk or protective but whether groups of factors are risk or protective. This would address the diversity and complexity of the phenomenon, leading to new risk assessment instruments and updates of current ones.

The knowledge of factors associated with IPF is essential for inclusion in risk assessment instruments. Their weights need to be pondered since not all have the same influence to predict homicides. Only one study of all included in the review has analyzed risk factor weights which is relative to the DA-I as mentioned in the results (Messing et al., 2013). There are certain studies that, although they do not directly display weights, could be extracted from the Odds Ratio (OR) and Relative Risk Ratio (RRR) scores reported statistically significantly. These are common measures of effect sizes that provide information on the probability that the homicide will occur, given particular factors. Factors with values of 1 indicate no effect on the homicide result, below one decreased risk and above one increased risk for this outcome in this (Schechtman, 2002; Andrade, 2015). The factors considered as low weight are partner control of women's life with OR ranged from 1.73 and 1.90 (Snider et al., 2009; López-Ossorio et al., 2021) and suicide ideas and attempts with RRR of 0.90 (Messing et al., 2020b) and OR of 1.994 (López-Ossorio et al., 2021). The last one could reduce the risk of IPF by a score lower than 1, being necessary to analyze it in future studies. There are two factors considered as medium weight, which is woman believe that man is capable of killing her by OR scores between 2.5 (Messing et al., 2017) and 5 (Snider et al., 2009) and battering during pregnancy by OR between 2.1 (Messing et al., 2017) and 3.4 (Snider et al., 2009). No high weight factors have been identified as there are controversies. In particular, escalation of violence has low weigh by an OR of 1.9 (Messing et al., 2017) but also high weight by an OR of 4.7 (Snider et al., 2009), violent and jealousy partner has a low weight by an OR ranged between 1.9 and 2.1 (Messing et al., 2017; López-Ossorio et al., 2021) and high weight by an OR of 5.5 (Snider et al., 2009), and strangulation has low weights by OR of 1.74 (Messing and Campbell, 2016) and 2.6 (Messing et al., 2017) and high weights by OR 4.1 (Snider et al., 2009). In this regard, it would be convenient to carry out studies on the weights of the mentioned factors and on those where this is not yet known. Moreover, it is crucial to also know the factors with RRR or OR scores lower than 1 to obtain information on elements that dampen the risk factors know up to know. This could improve homicide risk management by providing greater protection to cases with many risk factors and few protective factors.

The results also respond to the third research question, “what are the reliability and validity of the instruments?.” Some studies reported reliability, indicating that the instruments are 70% reliable and, consequently, 30% unreliable based on the consistency of the items for the outcome measured. This affirmation is extracted by a mean Cronbach's α score of 0.70 that is close to 1 (Campbell, 1986; Brown, 2002; Wang, 2015; Messing and Campbell, 2016; Messing et al., 2020b). The interrater reliability is only reported in one study, and it indicates good consistency of the instrument based on the degree of agreement on the items by an intraclass correlation coefficient of 0.83, which is close to 1 (Storey and Hart, 2014; Koo and Li, 2016). There is also one study that examined the construct validity, which indicates medium agreement with other similar measures due to a moderate positive correlation of 0.50 (Campbell, 1986; Adams et al., 2014; Chiu et al., 2015). The mentioned psychometric properties are not commonly revealed in studies included in the review, and future research should focus on these elements. The predictive validity is an exception due to these specific forms of criterion validity reported in most studies.

The balanced sensitivity and specificity scores of the instruments presented in the results do not always correspond to the prediction of IPF or attempted IPF but to specific cut-off scores and levels of danger. For instance, the updated version of the DA (Campbell et al., 2009; Storey and Hart, 2014) has a sensitivity of 0.55 and specificity of 0.97 for the extreme danger, while the balance is at the severe level with a sensitivity of 0.79 and specificity of 0.86. The consideration of extreme level is essential for predicting IPF or attempted IPF since it is closer to lethal outcome measured, although unbalanced. The same is applicable to the updated version of the DA-I (Messing et al., 2020b) which has a sensitivity of 0.29 and specificity of 0.969 for extreme levels compared to a sensitivity of 0.86 and specificity of 0.63 for severe levels. This data indicates underprediction contrary to the overprediction of the lethality screen (Messing et al., 2015a; Messing and Campbell, 2016) which has a high sensitivity of 0.93 and low specificity of 0.21. What is more, the DA-LE (Messing and Campbell, 2016; Messing et al., 2020b) has a balanced sensitivity of 0.60 and specificity of 0.70 approximately at the cut-off point of 7 over 11. It indicates low risk under that score and high risk above it, but as the cut-off increases, the sensitivity decreases and specificity increases significantly. For example, at a cut-off score of 9, the sensitivity is 0.24 and specificity is 0.90, and at 11, sensitivity is 0.04, and specificity is 0.98. The same is observed for the DA-5 (Snider et al., 2009), the updated version of the DA-5 (Messing et al., 2017), and the SIVIPAS (Echeburúa et al., 2009). These instruments could also lead to an underprediction of high-risk cases. In this regard, studies that assessed the PVW cases and then followed up on them to see whether the lethal or non-lethal result occurred report tool overprediction problems, whereas studies that analyzed know lethal and non-lethal PVW display underprediction problems (Campbell et al., 2009; Storey and Hart, 2014). It is necessary for future research to apply both types of studies to each of the instruments for IPF for stronger results.

The equilibrium of the mentioned elements is essential to discriminate high-risk cases from those that are not preventing a high number of false positives and false negatives. The first case makes it difficult to assist victims at risk because of the confusion generated by non-high-risk cases and the existence of limited resources, and the second case generates problems in the detection of high-risk cases and, therefore, in their prevention. The recently updated version of the DA (Messing et al., 2020a) overcomes this unbalanced limitation of the updated version of the DA (Campbell et al., 2009; Storey and Hart, 2014) due to an acceptable equilibrium in sensitivity and specificity for the extreme danger level. However, these measures should be tested again for the other mentioned studies.

In this section limitations of the included studies and future lines of research to address them have been presented. The process of systematic review also has several limitations that must be contemplated. First, it is possible to have additional studies related to risk assessment instruments for IPF, beyond those analyzed in the review. They have not been included because they are not in the databases used or didn't meet inclusion criteria such as not being in English or not being scientific articles. Second, the synthesis of the information from the studies has been carried out as neutrally and objectively possible, but there is the possibility that the interpretation of some results may not correspond with the original authors initially intended to transmit. The systematic review has also strengths. First, the systematic review has performed under the quality standards. The PRISMA guidelines (Page et al., 2021) was used allowing the course of the rigorous review process. Additionally, a quality assessment of the search strategy was conducted using an innovative text mining methodology LSA, and a quality assessment of the included studies through an adaptation of CASP, EPHPP, and CRD tools (Geraghty and Woodhams, 2015). Second, the review not only evidence the specific instruments for IPF but also detects their weaknesses and proposes future research to address them.

###  Practical Implications

The outcomes of this paper provide to first responders with specific evidenced-based knowledge on a wide range of risk assessment instruments for IPF and their psychometric properties. Furthermore, cultural and contextual adaptations as well as recent updates of the instruments are also reported. As a result, this research contributes to (1) simplify the choice of the appropriate instrument for professionals and (2) enhance the accuracy of prediction, since updated figures on reliability and validity are given. Last but not least, this knowledge facilitates the management of high-risk cases detected through interventions for preventing IPF.

## 5. Conclusion

The systematic review provides an overview of evidence-based risk assessment instruments for IPF, which are the Danger Assessment, the Danger Assessment for Immigrants, the Danger Assessment for Law Enforcement, the Danger Assessment-5, the Taiwan Intimate Partner Violence Danger Assessment, the Severe Intimate Partner Risk Prediction Scale, The Lethality Screen, and the H-Scale. Content of risk factors items of IPF, validity, and reliability of these instruments are synthesized. Information on country, year, sample, and data sources in which the studies that support these instruments were conducted is also summarized. This comprehensible knowledge could assist professionals involved in PVW use tools within an evidence-practice framework to identify women victims at risk of near-lethal or lethal violence by their partners and respond with risk mitigation strategies at a time to prevent it. In addition, this review highlights research gaps to be considered in future studies on this field.

## Data Availability Statement

The original contributions presented in the study are included in the article/supplementary material, further inquiries can be directed to the corresponding author.

## Author Contributions

EG-V, NA, FF-N, and DB-A have designed the systematic study. They have involved to determine the aims, the method and results configuration. EG-V and NA have searched for studies on databases and collaborated for the peer review. EG-V has drafted the manuscript and prepared the figures and tables with the NA, FF-N, and DB-A assistance. NA, FF-N, and DB-A have been responsible for supervising the paper. All authors contributed to results interpretation and critical discussion.

## Conflict of Interest

The authors declare that the research was conducted in the absence of any commercial or financial relationships that could be construed as a potential conflict of interest.

## Publisher's Note

All claims expressed in this article are solely those of the authors and do not necessarily represent those of their affiliated organizations, or those of the publisher, the editors and the reviewers. Any product that may be evaluated in this article, or claim that may be made by its manufacturer, is not guaranteed or endorsed by the publisher.
